# GENDER DIFFERENCES IN INFORMANT REPORTS OF COGNITIVE FUNCTIONING IN MEXICO AND THE US

**DOI:** 10.1093/geroni/igad104.1246

**Published:** 2023-12-21

**Authors:** Phillip Cantu

**Affiliations:** UTMB, Galveston, Texas, United States

## Abstract

**Background:**

Increasingly, researchers are attempting to measure cognitive functioning in large population-based surveys using both direct measurement and informant reports, but little work examines how gender affects measurements. For example, the role of gender in the division of labor is different in the U.S. and Mexico so questions relating to household chores will overestimate functional impairment in Mexico relative to the U.S. Method: Data from the U.S. Health and Retirement Study (HRS) and the Mexican Health and Aging Study (MHAS) will be used to examine disparities in how informants report cognitive change, measured by the total Community Screening Instrument for Dementia (CSI′D’) score, are related to gender of participants and informants. Result: Female participants in Mexico have higher CSID scores than men but not in the U.S. Conversely, female informants report higher CSID scores than male informants in the U.S. but not in Mexico.

**Conclusion:**

Further analysis is needed to determine if national differences in the association of gender and reports of cognitive impairment are due to differences in how informants report impairment.

